# Respiratory assessment and management of newborns and children with congenital lung diseases: a cohort study

**DOI:** 10.1186/s13052-025-01918-8

**Published:** 2025-03-11

**Authors:** Federica Porcaro, Antonella Coretti, Valerio Pardi, Ivan Pietro Aloi, Andrea Conforti, Francesca Petreschi, Renato Cutrera

**Affiliations:** 1 Pediatric Pulmonology & Cystic Fibrosis Unit, Respiratory Research Unit, Bambino Gesù Children’s Hospital, IRCCS, Rome, Italy; 2 General and Thoracic Surgery Unit, Bambino Gesù Children’s Hospital, IRCCS, Rome, Italy; 3 Neonatal Surgery Unit, Bambino Gesu’ Children’s Hospital, IRCCS, Rome, Italy

**Keywords:** Congenital lung disease, Malformations, Respiratory assessment, Children

## Abstract

**Introduction:**

Children with congenital lung disease (CLD) may suffer from long-term complications, such as impairments in lung growth, decreased total lung volume, recurrent lower respiratory tract infections and, in some cases, malignant transformation.

**Objective and methods:**

we described retrospective data on diagnostic process, clinical and functional data regarding a cohort of symptomatic and asymptomatic children with CLD followed in a single third level center in the last twenty years.

**Results:**

91 children were included in the study. Five classes of disease were examined. Bronchial tree and pulmonary abnormalities represent the most common anomalies. Despite the improved resolution of prenatal diagnosis, most of patients underwent chest CT scan to confirm the initial diagnostic suspicion. The most reported symptoms were wheezing, recurrent respiratory infections and acute respiratory failure. According to malformation type, patients underwent to surgery, endoscopic and/or medical treatment. Improvement of symptoms occurred faster in patients surgically and endoscopically treated. No statistical difference in the number of exacerbations before and after treatment was recorded, as well as no differences in spirometry values were observed among surgically and non-surgically treated children. No malignant transformation was observed in two patients with intra-lobar sequestration and hybrid lesion during the follow up period.

**Conclusion:**

the clinical presentation of congenital airway and lung disorders varies significantly depending on the type of malformation, making it challenging to standardize treatment strategies and follow-up programs. Based on our experience, prompt surgical or endoscopic intervention in early symptomatic children leads to faster symptom improvement and normal lung function in the follow-up period. However, further prospective studies are needed to better define optimal treatment strategies for these rare conditions, particularly for asymptomatic patients, for whom management approaches remain poorly established.

**Supplementary Information:**

The online version contains supplementary material available at 10.1186/s13052-025-01918-8.

## Introduction

Congenital lung disease (CLD) comprises a spectrum of developmental anomalies of the lung parenchyma, airways, vasculature or a combination of the above. Anyway, there are three other areas wherein malformation may affect the respiratory system including (1) the heart and great vessels, (2) the chest wall and (3) the abdomen [1]. Based on these premises and for the purpose of our study, we used a systematic approach and classified our patients affected by CLD using partially the classification proposed by Saleh and colleagues [2], but also considering patients with congenital diaphragmatic hernia (CDH).

All these malformations occur during embryogenesis because of abnormal organogenesis or dysregulation of cellular signalling within the epithelial–mesenchymal interaction [3].

Because of the paucity of good quality epidemiological studies, it is difficult to estimate the incidence of most considered abnormalities [1].

Recent advances in ultrasound imaging have increased the prenatal detection of CLD. Nevertheless, because they can be asymptomatic postnatally, the diagnosis may occur occasionally when imaging is made for other reasons.

Despite the advantageous opportunity to identify intrathoracic anomaly precociously with prenatal investigations, postnatal imaging remains crucial to characterize the lesion in view of possible surgical decisions.

Children with symptomatic CLD typically present non-specific respiratory symptoms, ranging from wheezing, stridor, recurrent respiratory infections, to respiratory distress episodes and acute life-threatening events. So, patients may be initially misdiagnosed and incorrectly treated [4].

Although surgical treatment is the gold standard for some symptomatic CLD (e.g. congenital pulmonary airway malformation), a consensus on asymptomatic cases has not been reached. Some clinicians recommend close monitoring of patients, while others advocate for elective resection of malformations like congenital pulmonary airway malformation (CPAM), intralobar pulmonary sequestrations, and bronchogenic cysts [5].

Anyway, it must be taken account that children with CLD may suffer from long-term complications, such as impairments in lung growth, decreased total lung volume, recurrent lower respiratory tract infections and, in some cases, malignant transformation [6].

In the present study we share our experience regarding patients affected by CLD and CDH, reporting information about type of malformation, diagnostic process to which they underwent to confirm diagnosis, and clinical and functional data recorded during the follow up period.

## Study design

This was a retrospective cohort study including children and adolescents affected by congenital lung disease and followed in the Pediatric Pneumology & Cystic Fibrosis Unit of Bambino Gesù Children’s Hospital in the last 20 years. Demographic, clinical, laboratory and instrumental data were extracted from the medical records, encompassing prenatal diagnosis, suspected malformation type, gender, age at symptoms onset, presenting symptoms, duration of symptoms prior to instrumental diagnosis, age at diagnosis, duration of symptoms before therapeutic intervention, variation in symptoms post-treatment initiation, airway microbial colonization, number of respiratory exacerbations, and results of pulmonary function tests. Written informed consent was taken from the patients or their parents for anonymously publication of data collected retrospectively during each assessment according to hospital procedures. The study was then approved by the Institutional Review Board of the Scientific Directorate of Bambino Gesù Children’s Hospital (RAP-2024-0001) before submission.

### Methods

Children and adolescents diagnosed with CLD in the last 20 years were included in the study. Our sample included patients primarily followed in our unit, those referred to our attention by the surgery department of our hospital in the pre- or post-operative period, and those referred to our center by other Italian hospitals.

Patients with airway abnormalities secondary to complete vascular rings, pulmonary artery sling (PAS), or associated to esophageal atresia were excluded from the present study.

For children suspected of CLD during the prenatal period, diagnosis confirmation was achieved through chest computed tomography (CT) scan with contrast enhancement and/or airway endoscopy. The choice between these investigations was made based on the diagnostic suspicion.

Typically, imaging for children with prenatal suspicion of cystic airway malformation was conducted within the first 3 months of life for clinically stable patients.

For cases identified postnatally, diagnosis was established due to recurrent respiratory symptoms or incidental findings during examinations made for other purposes.

During the follow up period, the repetition of imaging or endoscopic investigations was usually established according to the patient’s clinical conditions and the progression of symptoms following treatment.

As previously reported, five classes of diseases were examined, including: (1) static and (2) dynamic airway malformations, (3) bronchial tree and pulmonary abnormalities, (4) airway defects related to vascular anomalies (excluding vascular rings and pulmonary artery sling) and (5) diaphragm malformations (Table 1).

**Table 1:** Five classes of diseases examined and specific malformations

Classes of CLD	Malformation’s types	Numbers
Static airway anomalies	Subglottic stenosis,	2
	Tracheal stenosis,	5
	Bronchial stenosis	4
Dynamic airway anomalies	Tracheomalacia,	1
	Bronchomalacia	2
Bronchial tree and pulmonary anomalies	Bronchial and lobar agenesis,	6
	Bronchial atresia,	11
	Supernumerary bronchi,	1
	Tracheal bronchus,	6
	Bronchogenic cyst,	2
	Congenital lobar emphysema,	3
	Congenital pulmonary airway Malformation,	15
	Pulmonary hypoplasia,	8
	Pulmonary sequestration	4
Airway defects due to vascular anomalies	Pulmonary artery hypoplasia,	1
	Horizontal course of the anonymous artery	10
Diaphragm anomalies	Congenital diaphragmatic hernia,	8
	Diaphragmatic relaxation	2

Upon discovering a CLD, patients underwent additional investigations to identify concurrent malformations, such as cardiac and abdominal ultrasound examinations. Genetic evaluation was obtained for children with dysmorphic traits and /or multiple associated malformations.

Symptoms reported by patients and/or their parents were classified in respiratory and digestive ones. Respiratory exacerbation was defined by any of the following: change in cough quality from dry to wet and/or sputum production for ≥ 3 days, breathlessness, chest pain, crepitations, wheezing with or without an increase in values of infectious markers [7]. The definition derives from that use in patients with bronchiectasis, as it is not established for patients with airways malformations.

Sputum cultures were carried out during the follow-up visits for patients reporting mucous production. Microbial colonization was defined by the presence of two positive sputum culture separated by at least 3 months in 1 year without sign and symptoms of exacerbation [8].

Spirometry (Cosmed Quarck spirometer) was performed according to the criteria of the American Thoracic Society (ATS) and the European Respiratory Society (ERS) by all children aged > 6 years and adolescents able to cooperate [9]. Measurements included forced vital capacity (FVC) and forced expiratory volume in 1s (FEV1); values were expressed as percentage of the predicted normal values. Measurements were repeated 20 min after inhalation of 200–400 µg salbutamol metered dose inhaler (according to patient’s age) via a spacer. A positive bronchodilator response was defined as a FEV1 improvement > than 12% of baseline.

Treatment options varied depending on the type of disease and could include surgical, endoscopic, or medical interventions. Given that patients might have undergone multiple treatments, the intervention with the most significant impact was considered primary.

### Statistical analysis

Data analysis was performed using MedCalc ver. 15.8 (MedCalc software bvba).

The Shapiro–Wilk method was used to assess the normality of data. Continuous variables were described through means, standard deviations (SD), and medians whereas categorical variables were presented as frequency and percentages.

Categorical variables were compared with χ2 test. Cochran’s Q test was used to perform multiple comparison among groups.

One-way analysis of variance (ANOVA) was used to compare means, while Kruskal-Wallis was used for data not-normally distributed.

T test for independent sample used to compare two sample means from unrelated groups.

The Kappa coefficient was used to assess the agreement between prenatal and post-natal diagnosis.

A *p*-value below 0.05 was considered statistically significant.

## Results

### Demographic findings

A total of 91 children with CLD were included in the study, of whom 55 were male. Among them, 88 were Caucasian, while 3 were Asian. Patients came mainly from central and southern Italy. The median gestational age and neonatal weight were 38 weeks (interquantile range: 36–39) and 3410 gr (interquantile range: 2800–3382.5), respectively.

Among the enrolled patients, 11 (12%) had a family history of malformations, and 1 had consanguineous parents. Syndromic pictures were observed in 7 cases. These findings are summarized in Table 2.

**Table 2:** Demographic and clinical features of study’s population

Findings	Number (%)
**Total population**	91
**Male**	55 (60.4)
**Race**	
Caucasian	88 (96.7)
Asian	3 (3.3)
**Median gestational age (weeks)** ^∗^	38
**Median neonatal weight (gr)** ^&^	3410
**Prenatal diagnosis** ^$^	42 (46.15)
Morphological US	39
Heart US	4
Fetal MRI	2
Amniocentesis	3
**Syndromic pictures**	7 (7.7)
VACTER association	2
Beals syndrome	1
Goldenhar syndrome	1
Kabuki syndrome	1
Poland syndrome	1
Smith Maghenis syndrome	1
**Total number of types of disease** ^°^	91
Bronchial tree and pulmonary abnormalities	56 (61.5)
Airway static malformations	11 (12.1)
Airway dynamic malformations	3 (3.3)
Vascular anomalies	11 (12.1)
Diaphragm malformations	10 (11)

*Available for 85 patients

& Available for 81 patients

$ One patient has had more than one investigation

°One patient may have more than one malformation, but the malformation with the greatest clinical impact was considered the main

### Types of detected malformations

Patients were categorized into five classes as outlined in the methods section (Table 1). Bronchial tree and pulmonary abnormalities affected 56 (61.5%) children, while airway static and dynamic defects affected 11 (12.1%) and 3 (3.3%) patients, respectively. Additionally, vascular anomalies and diaphragm malformations affected 11 (12.1%) and 10 (11%) patients, respectively.

### Diagnostic investigations

Prenatal diagnostic information was available for 42 patients (46.2%). Thirty children (33%) received a diagnosis because symptomatic, while 17 (18.7%) were incidentally diagnosed; information was missing for 2 cases (2.1%).

With reference to prenatal diagnosis methods, 39 patients underwent morphological ultrasound (US), 4 had heart US, 2 underwent fetal magnetic resonance imaging (MRI), and 3 had amniocentesis; some of them had more than one investigation. Post-natal investigations included chest CT scan with contrast enhancement (71 cases), chest x-ray (6 cases), airway endoscopy (11 cases), chest (1 case) and heart US (1 case), as well as angiography (1 case). Chest CT scan was combined with airway endoscopy in 7 patients, with heart/chest US and angiography in the remain 3 cases.

There was no agreement between prenatal and post-natal investigations (weighted Cohen’s Kappa 0.000), with post-natal examinations demonstrating higher sensitivity compared to pre-natal ones (100% versus 88.10%).

The median age at instrumental diagnosis or confirmation for the entire population was 7 months. Among patient receiving diagnosis postnatally, bronchial tree and pulmonary abnormalities were diagnosed later compared to other classes. No statistical difference was observed in the age at instrumental diagnosis between groups diseases diagnosed postnatally.

### Symptoms

Symptoms reported by patients, or their parents encompassed both digestive and respiratory manifestations. Four children (4.4%) reported digestive symptoms (dysphagia and vomiting) other than respiratory ones. Respiratory symptoms were observed in a total of 66 patients (72.5%). Symptoms were significantly reported by patients diagnosed postnatally (*p* = 0.045), with a predominant occurrence noted in patients belonging to group 3 (*p* = 0.0165).

Wheezing, pneumonia, and acute respiratory failure were reported in 28.6%, 26.4%, and 16.5% of patients, respectively. Among reported respiratory symptoms, wheezing, recurrent respiratory infections, and acute respiratory failure reached statistical significance, as showed in Table 3.

**Table 3:** Respiratory complaints affecting study’s population

Symptoms*	Group 1	Group 2	Group 3	Group 4	Group 5	Total *n*. (%)	*p*-value
Acute respiratory failure	5	1	5	0	4	15 (16.5)	*0.0032*
Apnea	0	0	0	1	0	1 (1.1)	0.1183
Barky cough	1	0	0	1	0	2 (2.2)	0.1703
EIA	1	1	3	0	1	6 (6.6)	0.3197
Pleural effusion	1	0	0	0	0	1 (1.1)	0.1183
Pneumonia	4	1	15	3	1	24 (26.4)	0.7302
Pneumomediastinum	0	0	1	0	0	1 (1.1)	0.9594
Pneumothorax	0	0	2	0	0	2 (2.2)	0.8651
RRIs	4	0	2	2	1	9 (9.9)	*0.0150*
Respiratory distress at birth	1	0	2	1	2	6 (6.6)	0.3818
Stridor	1	1	0	3	0	5 (5.5)	*0.0009*
Wheezing	2	0	16	4	4	26 (28.6)	0.6007
No symptoms	1	0	23	1	0	25 (27.5)	*0.0078*

∗One patient may have more than 1 symptom

In total, 25 children (24.2%) were asymptomatic.

The median age at symptoms onset was 1 year of age for all 66 symptomatic patients, with no significant difference observed after subgroup analysis.

For 48 symptomatic patients for which information was available, the median interval between symptoms onset and the first diagnostic investigation was 0.44 years. However, diagnostic investigations were conducted before symptom onset in the remaining 18 symptomatic cases.

### Treatment strategy

Overall, 70 patients received treatment. Depending on the nature of the malformation, patients underwent surgery (44 cases, 48.4%), medical intervention (26 cases, 28.6%), or endoscopic procedures (5 cases, 5.5%). Five surgical treated patients received also medical (3/5) or endoscopic (2/5) interventions. Twenty-one patients did not receive treatment based on the type of abnormality and clinical presentation. There was no statistically significant difference in the distribution of treatment types among the five groups (Table 4). Among symptomatic patients treated after symptoms onset, the median interval between symptoms onset and the initiation of treatment was 0.41 years (24 patients) for those who underwent surgery, 0.55 years (2 patients) for those who underwent endoscopy, and 1.03 years (22 patients) for those who received medical treatment (inhaled beta2-agonist and/or corticosteroids, oral azithromycin, airways clearance techniques) (*p* = 0.0198). Considering patients receiving only one type of treatment (surgical, endoscopic, or medical), a statistical difference in the interval between symptoms onset and the timing of treatment was recorded for surgically managed patients belonging to group 3, as it occurred later compared to groups 4 and 5 (*p* = 0.0320).

**Table 4:** Distribution of treatment types among the five groups

Treatment	Group 1	Group 2	Group 3	Group 4	Group 5	Total (%)	*p*-value
Surgery	6	2	23	4	9	44 (48.4)	0.053
Endoscopy	1	0	2	2	0	5 (5.5)	0.304
Medical	3	0	15	4	4	26 (28.6)	0.687
None	1	1	17	2	0	21 (23)	0.1840

Please note: three and two surgical treated patients received also medical or endoscopic interventions, respectively

### Follow up period

Two patients were lost in the follow up during the study period. The median age at the start of follow up was 3.72 years, and at the end it was 8.95 years. The median duration of follow up for patients who completed the study was 3.17 years.

Overall, 49 children reported improvement in symptoms (fewer severe exacerbations and quicker resolution of acute infections), with one patient affected by bronchus suis showing improvement despite no treatment.

Among surgically, endoscopically, and medically treated patients, symptoms improved in 26, 2, and 20 patients, respectively. Improvement occurred after 1 months, 3 and 6.5 months after each treatment, respectively. The median age at symptoms improvement differed significantly among the three groups, being earlier in surgically and endoscopically treated patients (*p* = 0.000014).

Data on the mean number of respiratory exacerbations before and after treatment were available for 32 and 31 patients, respectively. However, no statical difference was observed. A similar analysis was conducted for 21 surgically treated patients, also yielding no statistical difference in the number of exacerbations before and after treatment.

Bacterial colonization was present in 10 patients (13.7%), primarily among patients in group 3. Haemophilus influenzae and Staphylococcus Aureus were the most found bacteria in airway secretions.

Data on respiratory function at 6 and 12 years were available for 32 and 17 children, respectively. No statistical difference was detected in median values of FEV1 and FVC at 6 and 12 years according to the type of malformation or when patients were grouped in surgically and non-surgically treated groups (Table 5).

**Table 5:** Median values of FEV1 and FVC compared using Kruskal Wallis test at 6 and 12 years. The total number of available respiratory function tests at 6 and 12 years was 32 and 17, respectively

DATA ACCORDING TO MALFORMATION’S TYPE					
	**Group 1**	**Group 3**	**Group 4**	**Group 5**	**p-value**
FEV1% at 6 ys	83.5	90.5	83	68	0.418
FVC% at 6 ys	85.5	91	86	79.5	0.697
FEV1% at 12 ys	93.5	99.75	71	94	0.832
FVC% at 12 ys	98	91	93	87	0.760
**DATA ACCORDING TO TREATMENT’S TYPE: SURGICAL OR NOT-SURGICAL TREATMENT**					
	**Surgical**		**Not surgical**		**p-value**
FEV1% at 6 ys	93		76.5		0.052
FVC% at 6 ys	91		85.5		0.121
FEV1% at 12 ys	93		67.5		0.203
FVC% at 12 ys	93		73		0.156

Legend: FEV1, forced expiratory volume in the first second; FVC, forced vital capacity; ys, years

Comparison of mean values of FEV1 and FVC at 6 and 12 years was possible for only 11 patients. Over time, no statistical difference was observed between the two measurements (FEV1 83% vs. 82.18%; FVC 89% vs. 88.54%).

At the end of the observation period, no malignant evolution was detected for 2 not surgically treated patients affected by pulmonary abnormalities (one with intra-lobar sequestration, and the other with hybrid lesion). Only one patient with CPAM type 1 developed malignancy in a different site from the pulmonary one (Wilms’ tumor), and genetic analysis revealed the presence of DICER1 gene mutation.

## Discussion

Our study collects a comprehensive range of congenital airway and lung disorders and describes the respiratory follow-up that patients underwent.

Bronchial tree and pulmonary abnormalities represent the most common anomalies, as this group contains malformations with the highest incidence compared to other groups [10, 11]. The widespread availability and improved resolution of prenatal ultrasonographic screening have certainly contributed to the early detection of these malformations [12]. However, prenatal diagnosis alone cannot guide the post-natal path, and more sensitive and specific diagnostic investigations are required to confirm the suspicious of CLD in the post-natal period. Indeed, more than half of our patients received diagnostic confirmation with chest CT scan with contrast enhancement. The superior anatomical characterization of the abnormalities essential for surgical plan, the speed of imaging acquisition, and the possibility to avoid sedation make chest CT scan with c.e. the gold standard for the postnatal evaluation of lung and airway development malformation [13–15].

We observed a higher sensitivity of post-natal examinations compared to pre-natal ones, in line with other studies on congenital lung malformations (CLMs) [16, 17]. This result was likely influenced by the greater number of malformations belonging to the third group. Indeed, it is known that CLMs become isoechoic to normal lung tissue late in gestation, which can mistakenly be considered as the disappearance of lesions [10]. Post-natal chest X-ray is commonly used as first diagnostic investigation to detect mediastinal shift or pneumothorax in newborn with pre-natal suspicion of CLM. Although this tool is valuable in newborn with unexplained respiratory failure, its use is questionable in asymptomatic patients with prenatally suspicion of CLM, because a normal chest x-ray does not rule out the diagnosis [18]. Therefore, given the toxicity derived by the repeated radiation exposure, lung ultrasound (LUS) can be considered as alternative approach to monitor infants with prenatal diagnosis of CLM. A single-center, retrospective cohort study suggested the routinary use of LUS for diagnosis and follow up of infants with prenatal suspicion of CPAM, given its high level of consistency with CT [19–21]. Anyway, despite increasing accuracy in prenatal evaluation and the availability of postnatal LUS, postnatal CT scan with c.e. remains necessary to confirm or exclude the initial diagnostic suspicion.

Regarding the age at instrumental diagnosis, we observed the group 3 was diagnosed later, and symptoms were more frequently reported in patients diagnosed in the post-natal period and belonging to group 3. This aligns with the findings of Lujan et al. [22], showing the subsequent development of symptoms in initially asymptomatic patients diagnosed with CPAM at birth and followed in long-term follow-up. In this case, the later onset of symptoms can be explained by the progressive hyperinflation of the affected lobe and the consequent atelectasis of the surrounding lung parenchyma until a mediastinal shift occurs.

As reported in the scientific literature, the clinical presentation of the malformations examined in our study is variable and non-specific, ranging from asymptomatic, to minimally or severely symptomatic, even at birth [23–27]. Although variability in symptomatology is expected given the different subgroups of disease, similarly to other studies [28–31], the most reported symptoms were wheezing, recurrent respiratory infections and acute respiratory failure: all these are typically favored by lumen reduction of the airway due to increased collapsibility of tracheal or bronchial airway cartilages or airway compression by vascular anomalies, as well as consequent abnormal mucus clearance.

Regarding treatment strategy, we detected a statistically significant difference in the interval between symptoms onset and timing of surgical treatment for patients in group 3, as it occurred later compared to groups 4 and 5. It is known that there are no standardized guidelines for the timing of resection in cases of bronchial tree and pulmonary abnormalities. Although older studies preferred postponing surgery until after the age of 12 months due to surgical and anesthetic risks [32, 33] more recent literature agrees in suggesting early surgical treatment for certain CLDs involving bronchial tree and pulmonary abnormalities (e.g. congenital pulmonary airway malformation). Good reasonings for earlier resection include preventing infections within the lesion that could complicate resection, avoiding the possibility of malignant transformation, and allowing compensatory growth of the adjacent lung parenchyma [34–37].

The potential worsening of symptoms severity within a very short time due to airway compression in group 4 and the displacement of hypochondriac organs into the thorax in group 5, justified the early and prompt surgical management in these two group of patients [27, 38]. For the other non-life-threatening situations and for those with unclear surgical indication, we always consider the possible transience of symptoms, severity, and impact on quality of life before deciding on surgery and its timing in collaboration with our thoracic surgeons.

During the short follow-up period, we recorded a faster improvement of symptoms in patients surgically and endoscopically treated, for which treatments allowed to eliminate evidently the cause of reported symptoms. Because the earlier onset of severe symptoms and consequently the need of a more invasive treatment, also the age at symptom’s improvement was lower for patients surgically and endoscopically treated compared to those only medically treated.

We didn’t detect a statistical difference in the number of exacerbations before and after treatment in our study population. Different results were described by other Authors. Elhattab et al. revealed that children with preoperative infection had higher rates of post-operative infection when treated for CLM [39]. Conversely, Markel et al. reported that children who underwent CLM resection experienced more frequent respiratory infections during the follow up period compared to the general population, concluding that resection does not eliminate the increased risk of pneumonia in this group of patients [40]. We found no similar studies investigating this topic for other classes of malformations.

Only 10 (11%) of our patients, mainly in group 3, presented bacterial colonization. Chest physiotherapy, often recommended by our team for most of the enrolled patients with a history of recurrent infections, could have led to this result thanks to the improvement of mobilization of airway secretions [41]. No studies investigating the effectiveness of respiratory physiotherapy for patients with bronchial tree and pulmonary abnormalities are available.

Regarding respiratory function, we observed that FEV1 and FVC values were within the normal range after surgery. These findings are supported by different reports showing normal lung function after surgery in the follow-up of CLMs [42, 43]. In addition, although patients who had surgery did not show statistical differences in spirometry values compared to non-surgically treated children, the surgically treated group exhibited better performance. Nir et al. also described no difference in FEV1 and FVC between operated and non-operated patients with vascular ring [44]. Conversely, Hijkoop et al. reported different results for patients followed for CLM (both surgically treated and untreated): in fact, the median FEV1 was significantly lower for patients undergoing surgery, while the other parameters did not differ significantly compared to non-treated patients [45]. This result could be affected by the study limitation of including only symptomatic patients. Nevertheless, in line with our experience, forced vital capacity was generally normal, supporting the hypothesis of the compensatory capacity of residual lung despite tissue loss and remodeling after surgical intervention [46].

In our cohort 2 patients with intra-lobar sequestration and hybrid lesion, not surgically treated due to parental decision, showed no signs of suspected malignant transformation by imaging after about 3 years of follow-up. Only one patient with CPAM developed Wilms’ tumor after 2 years of follow-up, but genetic analysis revealed the presence of a DICER1 gene mutation.

Pathogenic DICER1 variants were found in 70% of patients with all types of pleuropulmonary blastoma (PPB) [47] and have also been reported in other neoplasms [48]. Given the importance of this gene in tumors development, Schultz et al. recommended genetic testing and surveillance for DICER1-associated pulmonary and other tumors [49].

During our 20-year follow-up, we observed a gradual increase in the number of cases of CLD, likely due to advancements in the resolution of prenatal ultrasonographic screening. Whereas 20 years ago most CLD cases were diagnosed postnatally, in recent years we have seen a significant rise in prenatal diagnoses. Another notable change over time pertains to diagnostic procedures. In the past, following a prenatal diagnosis, the first postnatal examination typically involved a chest X-ray. However, to reduce unnecessary radiation exposure, chest CT scans with contrast enhancement have now become the standard first postnatal diagnostic test to confirm the diagnosis. As knowledge of DICER1 and its clinical implications has increased in recent years and given the described poor sensitivity of CT for identifying malignant lesions [50], our approach to pulmonary abnormalities has changed over time. In fact, we now test for DICER1 in all patients with lung cyst, especially if they are multi-septated, multiple, or bilateral, symptomatic (shortness of breath and pneumothorax due to cyst rupture) and prenatally unrecognized [47, 51].

We are aware that our study has several limitations. First, it is a single-institution retrospective study that includes patients who were not taken care of by our center at the time of birth, but subsequently because of the appearance of symptoms or for a second opinion. Second, the lack of standardized protocols for prenatal and post-natal diagnostic and therapeutic interventions: these protocols often vary based on the experience of the initial treatment center. In addition, the rarity of the reported diseases and the variability in clinical presentation influenced diagnostic and therapeutic decisions. Third, there is a selection bias due to the heterogeneity of reported diseases and the larger sample size of group 3, compared to the others. Fourth, it is possible that not all patients with CLD evaluated at our center are referred to our Pulmonology service, especially if asymptomatic, potentially underestimating the true number of cases. Lastly, the follow-up period remains relatively short, limiting our ability to evaluate the long-term risk of malignancy. On the other hand, our study has several strengths: (1) the inclusion of a wide range of disorders affecting lung parenchyma, airways and vascular development in a large sample; (2) the inclusion of both symptomatic and asymptomatic patients, and (3) standardized assessments during follow-up.

## Conclusions

Although surgical treatment is considered the gold standard for some symptomatic CLDs and CDH, there is still no consensus on a well-established management strategy for asymptomatic CLDs. This gap is likely due to the paucity of long-term studies on the infectious risk, lung function and risk of malignancy in this type of disease.

Further prospective studies reporting multidisciplinary assessments beginning in the post-natal period and continuing over time during pediatric age are advisable for both symptomatic and asymptomatic CLD patients to provide proper answer to these crucial questions.

## Electronic supplementary material

Below is the link to the electronic supplementary material.

Supplementary Material 1

Supplementary Material 2

## Data Availability

The datasets used and/or analyzed during the current study are available from the corresponding author on reasonable request.

## Abbreviations

CLD: Congenital lung disease
CDH: Congenital diaphragmatic hernia
CPAM: Congenital pulmonary airway malformation
PAS: Pulmonary artery sling
CT: Computed tomography
ATS: American Thoracic Society
ERS: European Respiratory Society
FVC: Forced vital capacity
FEV1: Forced expiratory volume in 1s
SD: Standard deviations
US: Ultrasound
MRI: Magnetic resonance imaging
CLMs: Congenital lung malformations
LUS: Lung ultrasound
PPB: Pleuropulmonary blastoma
