# Supplementary material for: Respiratory assessment and management of newborns and children with congenital lung diseases: a cohort study
Source: Ital J Pediatr. 2025 Mar 11;51:71. doi: 10.1186/s13052-025-01918-8 (PMC11899023; doi:10.1186/s13052-025-01918-8)
Supplement: Supplementary file 1 — Supplementary Material 1 [file 13052_2025_1918_MOESM1_ESM.docx]

Reviewer #1: In this study the authors report their 20-year experience regarding 91 patients affected by congenital lung disease (CLD) and congenital diaphragmatic hernia (CDH), providing information about type of malformation, diagnostic process, and clinical and functional respiratory data at follow up.
The topic is interesting given the relative rarity of these congenital conditions. However, the document has numerous flaws that could compromise its value.
General comments
- Main limitations of this study include the single center, the retrospective design, and lack of standardized protocols for prenatal and postnatal diagnostic and therapeutic interventions, apparently left to discretional judgment of the healthcare providers.
- Even if the study population is relatively large, it was distributed in 20 years thus implying that the authors observed about less than five cases per year. In addition, the panel of reported diseases was very heterogeneous, making comparisons very difficult.
- the authors' decision to divide the population into five disease classes appears to be arbitrary, as the classification of Saleh et al. (ref.#2) is substantially different. In particular, it is unclear why static and dynamic airway abnormalities were kept distinct, given the small size of group 1 (only three patients, apparently).
- it is not well specified whether the patients were taken care of by the center at the time of birth or subsequently. If so, it would be appropriate to provide further details.
-there is no information on any changes over time in terms of the type of pathologies, diagnostic procedures and treatment strategies. Perhaps it would be interesting for the authors to describe what the most relevant developments have been in this regard.
- the manuscript needs a careful English editing.

Dear Reviewer,

Thank you for your thoughtful comments. We have addressed your concerns as follows.

Several of your queries were already discussed in the text, where we outlined the limitations of our study. Nonetheless, we have added clarifying points to address additional aspects.

*“We acknowledge that our study has several limitations. First, it is a single-institution, retrospective analysis, which includes patients who were not taken care of by our center at the time of birth, but subsequently because of the appearance of symptoms or for a second opinion. Second, there is a lack of standardized protocols for prenatal and postnatal diagnostics and therapeutic interventions. These protocols often vary based on the experience of the initial treatment center. The rarity of the reported diseases and the variability in clinical presentation influenced diagnostic and therapeutic choices. Third, a selection bias exists due to the heterogeneity of the diseases studied, along with an uneven sample size, particularly for group 3, which was larger than the others. Fourth, it is possible that not all patients with CLD evaluated at our center are referred to our Pulmonology service, especially if asymptomatic, potentially underestimating the true number of cases. Lastly, the follow-up duration remains relatively short, limiting our ability to evaluate long-term risks, such as malignancy.”*

Regarding the initial diagnostic uncertainty, we faced challenges in three specific cases. For these patients, initial differential diagnoses were as follows:

- Patient 1: suspected congenital airway pulmonary malformation (CAPM)
- Patient 2: suspected congenital lobar emphysema
- Patient 3: suspected pulmonary sequestration

Subsequent chest CT scans during follow-up allowed us to confirm the final diagnoses: lobar agenesis, bronchomalacia with resultant air trapping, and lobar sequestration, respectively. Despite the diagnostic uncertainty, our follow-up approach remained consistent. Decisions regarding repeat CT scans were driven by symptom progression or any reported worsening.

We also emphasized in the manuscript that our approach to managing pulmonary abnormalities has evolved. Specifically:

*“…*During our 20-year follow-up, we observed a gradual increase in the number of cases of CLD, likely due to advancements in the resolution of prenatal ultrasonographic screening. Whereas 20 years ago most CLD cases were diagnosed postnatally, in recent years we have seen a significant rise in prenatal diagnoses. Another notable change over time pertains to diagnostic procedures. In the past, following a prenatal diagnosis, the first postnatal examination typically involved a chest X-ray. However, to reduce unnecessary radiation exposure, chest CT scans with contrast enhancement have now become the standard first postnatal diagnostic test to confirm the diagnosis. As knowledge of DICER1 and its clinical implications has increased in recent years and given the described poor sensitivity of CT for identifying malignant lesions(50), our approach to pulmonary abnormalities has changed over time. In fact, we now test for DICER1 in all patients with lung cyst, especially if they are multi-septated, multiple, or bilateral, symptomatic (shortness of breath and pneumothorax due to cyst rupture) and prenatally unrecognized(47,51)*.”*
Specific comments
- Title: because the paper is reporting diagnostic, therapeutic and follow-up information, maybe I would change the title as follows or with a similar title: Respiratory assessment and management of newborns and children with congenital lung diseases: a cohort study.

Dear Reviewer, thank you for suggesting improvements to our title. We have made the changes.
- Abstract: "The most reported symptoms were stridor, recurrent respiratory infections and acute respiratory failure." This statement is not entirely supported by the data presented (see results and discussion). For example, stridor was reported in only 5.5%, apparently.

Dear Reviewer, we apologize for the mistake; we have corrected it.
- Abstract conclusions should be more related to the reported findings.

Congenital airway and lung disorders are rare conditions that require postnatal investigations to better characterize the type of lesion, even if there is an initial prenatal suspicion. The clinical presentation varies significantly depending on the type of malformation, making it challenging to standardize treatment strategies and follow-up programs. Based on our experience, prompt surgical or endoscopic intervention in early symptomatic children leads to faster symptom improvement and normal lung function in the follow-up period. However, further prospective studies are needed to better define optimal treatment strategies for these rare conditions, particularly for asymptomatic patients, for whom management approaches remain poorly established.

- Introduction: please check the order in which the bibliographical references appear.

Dear Reviewer, we have reviewed the order of the bibliographical references and confirm that they are correctly arranged.
- Pag. 4, line 78: please add some references.

Dear Reviewer, we have added the follow reference: Bush A. Rare Lung Diseases: Congenital Malformations. Indian J Pediatr. settembre 2015;82(9):833–40.
- Pag. 5, line 100: "Patients with airway abnormalities secondary to complete vascular rings, pulmonary artery sling 101 (PAS), or associated to foregut malformations were excluded from the present study." It is not clear why these conditions were excluded. Furthermore, I am not sure if bronchogenic cysts and pulmonary sequestrations are actually included among foregut malformations. Please check.

Dear Reviewer in our clinical practice, we dedicate outpatient clinics for patients with *primary* abnormalities of airways, lung and great vessels, patients with airway abnormalities *secondary* to complete vascular rings or associated to PAS (although PAS is not classically defined as complete vascular ring, it acts like a ring), and those with airway abnormalities *associated to* esophageal atresia. For this study, however, we included only *primary* defects. As reported in Table 1, bronchogenic cyst and pulmonary sequestration are included in our sample.
- Page 5, line 112: "As previously reported, using a systematic approach, five classes of diseases were examined.." Please explain better which systematic approach was used and why five classes of diseases were chosen.

Dear Reviewer, thank you for the opportunity to clarify the rationale behind our classification.

As mentioned in the Introduction, we “partially adopted the classification proposed by Saleh and colleagues” for patients affected by CLD. While, as you observed, Saleh et al.'s classification differs and encompasses broad categories, such as tracheal and bronchial branching anomalies, lung bud malformations, and vascular abnormalities, our approach was to follow a similar framework. However, for greater clarity, we chose to distinguish between anatomical and functional tracheal alterations and to include lung hypoplasia due to CDH as a separate category, given that CDH represents a distinct clinical entity.

We acknowledge that our classification is somewhat arbitrary, and it is not our intention to propose a new classification system.

- Respiratory exacerbations were defined by applying methods used for patients with bronchiectasis, a population substantially different from that reported in the present study. I wonder if the authors should have developed criteria that were more specific and suited to the study patients, even if not standardized.

Dear Reviewer, we choose to use standardized criteria similar to those used for patients with bronchiectasis because these criteria aligned well with the exacerbation episodes observed in our populations.
- Pag. 7, line 159: I would suggest to add a table showing more in details types and numbers of all malformations included in the study

Dear Reviewer, types of malformations were already available in Table 1. As you suggested, we have added also the number.
-  Pag. 7, line 159:"A significant statistical difference was noted in bronchial tree and pulmonary abnormalities...". I am not sure that statistical analysis is appropriate for this merely descriptive section.

Dear Reviewer, we have modified the text reporting descriptive statistic.
- Pag. 8, line 175: Please explain more clearly what agreement you are alluding to with this sentence.

Dear Reviewer, when there are two evaluators and the evaluations are qualitative, the most widely used index for measuring concordance is the Kappa Coehn coefficient. Therefore, we used it, because it measures the degree of real agreement between the two evaluations.
- Pag. 8, line 179: Again, statistical analysis makes sense in this section. Numbers are too little and the incidence of each malformation is not clearly defined.

Dear Reviewer, we have deleted the sentence.
- Pag. 8, line 182: "Among patient receiving diagnosis postnatally, bronchial tree and pulmonary abnormalities were diagnosed later compared to other classes". What the authors mean with "later"?

Dear Reviewer, we mean that diagnosis was made at a later age (median age 5.1 years).

- Pag. 8, line 187: "..digestive symptoms...". please specify.

Dear Reviewer, digestive symptoms included dysphagia and vomiting (we have added the information in the text).
- Pag. 8, line 192: "Nevertheless, only stridor, recurrent respiratory infections, and acute respiratory failure reached statistical significance, as showed in Table 3." This statement is not appropriate given that each subgroup of disease is expected to present different symptoms and numbers are too limited. In addition, Table 3 should report the absolute numbers (and percentage), specifying which item was statistically different from another, ideally then providing some interpretation in the discussion, if appropriate.

Dear Reviewer, we have modified the sentence and Table 3 as you suggested.
- Pag. 9, line 198 and 209: please check consistency (days, month, fraction of years..).

Dear Reviewer, we have modified the text.
- Pag. 9, line 210: "..for those who received medical treatment..": Could you specify which sort of treatments were provided to these patients?

Dear Reviewer, patients received inhaled beta2-agonist and/or corticosteroids, oral azithromycin, and airways clearance techniques like maintenance treatment based on their clinical pictures. Please, see the modified text.
- Pag. 10, line 219: How was the improvement of symptoms measured or quantified?

Dear Reviewer, parents reported an improvement in symptoms reflected by fewer severe exacerbations and quicker resolution of acute infections. We have added the information in the text.
- Pag. 10, line 223: How the median age at symptom improvement was determined? By history taking or actual follow-up visits?

Dear Reviewer, the information was extrapolated by the review of the documents of follow up visits during which the timing of improvement was recorded.
- Pag. 11, line 262: I would add a few comments about the potential role of standard chest-X-ray and thoracic ultrasound scan in the post-natal phase, given their scarce invasive impact.

Dear Reviewer, we have added comments about the topic you suggested.

- Pag. 12, line 272: ".. the most reported symptoms..": see my previous comments. Stridor was not the most reported symptom, according to the presented data (text and Table 3). Wheezing was present in 28.6% of patients..

Dear Reviewer, we have corrected the text.
- Pag. 13, line 288: " ..dysplacement of hypochondriac organs in the mediastinum..) Did you mean thorax..?

Dear Reviewer, we have made the changes in the text.
- Pag. 14, line 327 onward: the observation on DICER 1 is interesting, but maybe eleven lines about this point are too many.

Dear Reviewer, we acknowledge that we may have written extensively about the DICER1 gene. However, we believe it is important to emphasize its clinical value in identifying lesions at risk for neoplastic disorders. Nonetheless, we have made an effort to condense the section dedicated to the DICER1 gene.

- Pag. 15, line 338: Limitations should be better specified, including those related to the points raised above.

Dear Reviewer, we have specified the limitations as you suggested.
- Table 1 is not easy to read. more detailed information should be provided (numbers...)

Dear Reviewer, we have modified the Table 1.
- Table 3: maybe I would add a row in this table, at the bottom, including a "No symptoms " subheading.

Dear Reviewer, we have added the row “No symptoms”.
- Table 4: I would add a fourth subheading in this table, namely "None", as several patients did not receive any treatment, and this information could be interesting for the reader.

Dear Reviewer, we have added the information in Table 4.
- Table 5: were patients belonging to Group 2 not investigated?

Dear Reviewer, we hadn’t spirometry information about the three patients belonging to group 2.
- Figure 1 should be removed, as it probably does not provide useful information for an international audience.

Dear Reviewer, we have removed the Figure 1 as you suggested.

Reviewer #2: The study is well designed and written
It is of interest to doctors working in the field of neonatology, pediatric respiratory and pediatric surgery
Tables are well representative of the study
It is also positive to mention the limitations and the strong points of the study at the end of the discussion.

Dear Reviewer, thank you for your positive comments.
