# Supplementary material for: Respiratory assessment and management of newborns and children with congenital lung diseases: a cohort study
Source: Ital J Pediatr. 2025 Mar 11;51:71. doi: 10.1186/s13052-025-01918-8 (PMC11899023; doi:10.1186/s13052-025-01918-8)
Supplement: Supplementary file 2 — Supplementary Material 2 [file 13052_2025_1918_MOESM2_ESM.docx]

Dear Editor,

We are pleased to submit our paper entitled “**Respiratory assessment in children with congenital lung disease: a cohort study”**, so that it can be considered for publication by the Italian Journal of Pediatrics.

Because children with congenital lung disease (CLD) may suffer from long-term complications (impairments in lung growth and function, recurrent lower respiratory tract infections and, in some cases, malignant transformation), we would like to share our experience regarding symptomatic and asymptomatic patients affected by CLD, reporting information about type of malformation, diagnostic process to which they underwent to confirm diagnosis, treatment and clinical and functional data recorded during the follow up period.

Because for some conditions there is no unique management, we hope that shared information can help improve management of this group of patients.

The manuscript has not been published previously and is not under consideration elsewhere.

All authors are responsible for reported research and have approved the manuscript as submitted. They don’t have any conflict of interest to disclose.

Best regards

Federica Porcaro
